# ICIs联合治疗应用于*EGFR*突变靶向治疗耐药后非小细胞肺癌的研究进展

**DOI:** 10.3779/j.issn.1009-3419.2023.101.17

**Published:** 2023-05-20

**Authors:** LI Jiamo, YAO Xingyu, QIU Longjue, ZHANG Ru, WANG Gang

**Affiliations:** 116001 大连，大连大学附属中山医院肿瘤科; Department of Oncology, Zhongshan Hospital Affiliated to Dalian University, Dalian 116001, China

**Keywords:** 肺肿瘤, 免疫检查点抑制剂, 表皮生长因子受体, 靶向治疗, Lung neoplasms, Immune checkpoint inhibitors, Epidermal growth factor receptor, Targeted therapy

## Abstract

随着肺癌精准医学的发展，靶向治疗极大地改善了晚期非小细胞肺癌（non-small cell lung cancer, NSCLC）患者的生存和预后，但获得性耐药的发生使患者最终面临无靶向药可用的局面，对于这部分患者后续没有标准的治疗选择。免疫检查点抑制剂（immune checkpoint inhibitors, ICIs）的出现使晚期NSCLC治疗发生革命性的改变，但由于表皮生长因子受体（epidermal growth factor receptor, EGFR）突变的NSCLC具有免疫抑制的肿瘤微环境（tumor microenvironment, TME）等独特之处，使得单一的ICIs治疗在EGFR突变的NSCLC患者中临床获益有限，将ICIs与化疗和/或靶向等治疗相结合是大势所趋。该篇综述中进一步讨论了可能从ICIs治疗中受益的具有EGFR突变的潜在亚群，还分析了在联合免疫治疗时代下如何决策可使ICIs治疗应用于EGFR突变靶向治疗耐药后NSCLC患者获得疗效最大化，以期实现个体化治疗。

## Abstract (en)

With the development of precision medicine for lung cancer, targeted therapy has greatly improved the survival and prognosis of patients with advanced non-small cell lung cancer (NSCLC), but the occurrence of acquired drug resistance ultimately leads to patients with no targeted drugs available and no standard treatment options for this group of patients afterwards. The emergence of immune checkpoint inhibitors (ICIs) has revolutionized the treatment of advanced NSCLC. However, due to the unique features of NSCLC with epidermal growth factor receptor (EGFR) mutation, such as immunosuppressive tumor microenvironment (TME), single ICIs treatment has limited clinical benefits in NSCLC patients with EGFR mutation, and the combination of ICIs with chemotherapy and/or targeted therapies is the trend. This review further discusses potential subpopulations with EGFR mutations that may benefit from ICIs treatment, and analyzes how decisions can be made in the era of combined immunotherapy to maximize the efficacy of ICIs treatment in EGFR mutation targeted therapy for NSCLC patients with drug resistance, with the aim of achieving individualized treatment.

## 1 前言

根据世界卫生组织（Word Health Organization, WHO）报告的数据^[1]^，2020年有180万例患者死于肺癌，肺癌仍是常见的癌症死亡原因之一。非小细胞肺癌（non-small cell lung cancer, NSCLC）占所有肺癌患者的80%-85%，其中10%-50%的NSCLC患者存在表皮生长因子受体（epidermal growth factor receptor, EGFR）突变，如ex19del或ex21 L858R错义突变。肺癌驱动基因阳性的发现使肺癌的治疗转向个性化靶向治疗时代，EGFR酪氨酸激酶抑制剂（tyrosine kinase inhibitors, TKIs）为EGFR突变阳性患者提供了更高的客观缓解率（objective response rate, ORR）和无进展生存期（progression-free survival, PFS）^[2,3]^。美国临床肿瘤学会（American Society of Clinical Oncology, ASCO）、欧洲医学肿瘤学会（European Society of Medical Oncology, ESMO）和美国国立综合癌症网络指南将EGFR-TKIs作为EGFR突变晚期NSCLC患者的一线治疗^[4]^。尽管EGFR-TKIs具有良好和持久的治疗反应，但获得性耐药限制了EGFR-TKIs的长期疗效，大多数患者会对EGFR-TKIs产生耐药，最终发展为疾病进展（progressive disease, PD）^[5]^，含铂类药物的化疗仍是TKIs治疗耐药后患者的首选治疗方案，但中位PFS（median PFS, mPFS）仅为4个月-5个月^[6]^，所以这部分患者急需有效的个体化治疗手段。本研究旨在针对EGFR-TKIs耐药后应用免疫联合其他药物治疗的相关研究进行综述，并进一步讨论可能从免疫检查点抑制剂（immune checkpoint inhibitors, ICIs）治疗中受益的具有EGFR突变的潜在亚群，以期为临床及科学研究提供参考。

## 2 EGFR-TKIs耐药后的相关免疫机制

近年来，针对细胞毒性T淋巴细胞抗原-4（cytotoxic T lymphocyte associated antigen-4, CTLA-4）、程序性细胞死亡蛋白1（programmed cell death-1, PD-1）或程序性细胞死亡配体1（programmed cell death-ligand 1, PD-L1）的ICIs的使用已成为癌症治疗领域中最有前途的方法之一。对于NSCLC患者尽管手术切除、放射治疗、以铂类为基础的化疗等方案治疗是可行的，根据分期和地区差异其5年生存率仅为4%-17%不等^[7]^。几种针对PD-1（帕博利珠单抗、西米普利单抗）或PD-L1（纳武利尤单抗、阿替利珠单抗）的ICIs已被美国食品药品监督管理局（Food and Drug Administration, FDA）批准用于晚期NSCLC一线的临床治疗，而且在临床实践中体现出显著的疗效^[8⇓-10]^。

ICIs显著改变了晚期NSCLC的治疗模式，但目前相关研究得出ICIs并不适合所有患者，治疗效果主要取决于一些分子生物标志物，如PD-1、PD-L1、肿瘤突变负荷（tumor mutation burden, TMB）、CD8^+ ^T细胞浸润、T淋巴细胞免疫球蛋白黏蛋白-3（T cell immunoglobulin domain and mucin domain-3, TIM-3）等^[11]^，其应答率也与肿瘤微环境（tumor microenvironment, TME）密切相关^[12]^。有研究^[11,13,14]^也进一步揭示了ICIs在EGFR突变的NSCLC患者中的临床获益有限的耐药机制，从ICIs预测指标来看，这部分患者无论TMB还是PD-L1均明显低于野生型患者；另一方面，致癌EGFR信号通路可以通过重塑TME引发免疫逃逸，特别是无炎症表型、主要组织相容性复合体I类缺失、CD73表达上调T细胞克隆性降低的肿瘤。此外，研究^[14]^表明经过TKIs治疗后TME会被激活，如增加树突状细胞和CD8^+^细胞使得调节性T细胞（regulatory T cell, Treg）减少并抑制M2样巨噬细胞极化；其次PD-L1的表达在TKIs治疗期间会发生波动，并在PD后发生变化。一项配对分析的研究^[15]^表明，患者在经过TKIs治疗耐药后，肿瘤细胞阳性比例分数（tumor cell proportion score, TPS）≥50%的数量从14%增加到28%，特别是在EGFR T790M阴性患者中尤为明显^[15,16]^。相关研究^[15,17]^表明不同EGFR-TKIs的耐药机制存在核因子kappa B（nuclear factor kappa B, NF-κB）及丝裂原活化蛋白激酶（mitogen-activated protein kinase, MAPK）、磷脂酰肌醇-3激酶-蛋白激酶B（phosphoinositide-3 kinase-protein kinase B, PI3K-Akt）、激活蛋白-1（activator protein-1, AP-1）信号通路的参与从而诱导PD-L1表达上调使得肺癌的免疫逃逸得以促进。

## 3 免疫单药治疗应用于EGFR-TKIs耐药后的局限性

虽然很多研究^[11⇓⇓⇓⇓-16]^为ICIs应用于治疗EGFR-TKIs耐药后的NSCLC患者提供了理论基础，但ICIs单药作为EGFR突变的NSCLC二线治疗获益仍非常有限。CheckMate 153是一项多中心IIIB期/IV期临床研究^[18]^，纳入既往经治的转移性NSCLC患者接受纳武利尤单抗治疗，其中EGFR突变亚组疾病控制率（disease control rate, DCR）仅为11%（n=55），而EGFR野生型亚组DCR为16%（n=300）。一项汇总了CheckMate 057、KEYNOTE-010和POPLAR研究的荟萃分析^[11]^，评估了纳武利尤单抗、帕博利珠单抗及阿替利珠单抗二线治疗EGFR突变晚期NSCLC的疗效。研究结果显示，与多西他赛相比，在EGFR突变的患者中接受ICIs治疗的患者并没有改善总生存期（overall survival, OS），差异也无统计学意义[风险比（hazard ratio, HR）=1.05，95%CI：0.70-1.55，P<0.81]。另一项系统回顾和荟萃四项分析（加入了OAK研究）^[11]^也得出了同样的结论。在2021年ASCO会议上公布的WJOG8515L试验^[19]^结果表明，无论PD-L1表达如何，纳武利尤单抗的疗效远远不及化疗（ORR：9.6% vs 36%；DCR：38.4% vs 76%；mPFS：1.7个月 vs 5.6个月）。

虽然研究^[11,18]^提示EGFR突变亚群有不理想的后线免疫治疗效果，但ATLANTIC研究^[20]^或能部分改变这种认识。ATLANTIC研究^[20]^是一个多中心、开放、单臂II期临床研究，该研究探讨了度伐利尤单抗三线或后线治疗晚期NSCLC的疗效及安全性，该研究也第一次前瞻性评估了ICIs在EGFR/间变性淋巴瘤激酶（anaplastic lymphoma kinase, ALK）阳性患者的疗效。其中1队列（EGFR/ALK+）中有12.2%（9/74）的患者达到客观缓解，且1队列的一项探索性分析中发现，肿瘤细胞（tumor cell, TC）≥25%患者中，所有达到客观缓解的患者均为EGFR+患者。EGFR+且TC≥25%亚组患者中mOS为16.1个月。BIRCH研究^[21]^是以阿替利珠单抗单药作为一线、二线或后线治疗NSCLC的II期单臂试验，结果显示不论是否存在EGFR或鼠类肉瘤病毒癌基因（kirsten rat sarcoma viral oncogene, KRAS）突变均有不同程度的获益，其中PD-L1高表达的EGFR突变患者的ORR为31%。以上结果提示EGFR-TKIs耐药患者仍可尝试应用ICIs，但ICIs单药治疗反应率还是较低的，需要探索更优化的免疫联合治疗方案。

## 4 免疫联合治疗应用于EGFR-TKIs耐药后的探索

### 4.1 ICIs联合EGFR-TKIs治疗

据研究^[11,22,23]^报道，在NSCLC的小鼠模型中，EGFR突变诱导EGFR信号通路激活PD-L1表达并诱导肿瘤逃逸的发生，这表明ICIs与EGFR-TKIs的联合治疗可能是一种有希望的治疗策略。众所周知，ICIs介导的不良反应通常被称为免疫相关不良事件（immune-related adverse events, irAEs），导致其发生的具体原因尚不清楚，目前考虑为免疫平衡的破坏引起^[24]^。CheckMate 012^[25]^是一个多队列的I期研究，旨在评估纳武利尤单抗联合不同药物（包括厄洛替尼）治疗晚期NSCLC，其中入组21例患者（20例既往接受过厄洛替尼且出现PD，1例既往未接受过TKIs治疗），该队列ORR为15%，mPFS和mOS分别为5.1个月（95%CI: 2.3-12.1）和18.7个月（95%CI: 7.3-NR）。安全性评估：所有患者均出现至少1次治疗相关性不良事件（treatment-related adverse events, TRAEs），大多数为1级-2级，5例患者出现3度TRAEs，11例患者因为irAEs导致治疗延迟，2例患者出现治疗中断。GEFTREM^[26]^是一项多中心、开放标签的I期临床研究，纳入EGFR突变且接受一代TKIs类药物治疗后进展的患者，接受标准剂量的吉非替尼联合替西木单抗，替西木单抗分为3个剂量（3 mg/kg、6 mg/kg、10 mg/kg），主要研究终点为安全性、耐受性，以建立II期推荐给药剂量。共纳入27例患者，其中4例出现剂量限制性毒性。3度及以上不良反应发生率为81%，72%的患者疗效评估为疾病稳定（stable disease, SD）、完全缓解（complete response, CR）或部分缓解（partial response, PR），mPFS为2.2个月，患者均终止了药物治疗。CAURAL研究（NCT02454933）^[27]^主要评估了奥希替尼联合度伐利尤单抗对比单独使用奥希替尼应用于接受过TKIs治疗的T790M阳性NSCLC患者的疗效和安全性差异。在另外一项单独的IB期Tatton试验（NCT02143466）^[4]^中，该研究旨在评估奥希替尼联合度伐利尤单抗治疗EGFR突变晚期NSCLC的安全性和有效性，然而由于奥希替尼联合度伐利尤单抗治疗组的间质性肺疾病（interstitial lung disease, ILD）发生率高达38%，导致该研究联合治疗队列招募提前终止，CAURAL招募也提前终止。有研究^[22]^结果显示，奥希替尼单药组对比联合组ORR分别为80%和64%。虽然CAURAL研究入组患者数量有限，但与奥希替尼单药治疗相比，度伐利尤单抗和奥希替尼联合治疗组没有治疗效果的明显提升。在厄洛替尼联合阿替利珠单抗的IB期研究中，入组EGFR阳性初治局部晚期或晚期转移性NSCLC患者，虽然ORR达到75%，mPFS为11.3个月。但3级-4级不良事件的发生率为39%，其中谷丙转氨酶（alanine aminotransferase, ALT）升高占7%，发热占7%，皮疹占7%，中断阿替利珠单抗治疗的患者约为18%^[27]^。这些研究^[26,27]^表明，虽然从理论上讲ICIs联合TKIs治疗是有希望的，但是一些联合治疗的早期试验即出现意想不到的高不良事件发生率，并且反应率的提升也是有限，目前研究结果不推荐ICIs联合TKIs治疗应用于临床。

### 4.2 ICIs联合化疗

ICIs与化疗联合治疗的方法已成为一个主要研究方向，同时ICIs联合以铂类为基础的化疗也是目前推荐的晚期驱动基因阴性NSCLC患者的一线治疗方案^[5]^。细胞毒性化疗药物通过影响PD-L1表达、TMB和抗肿瘤免疫活性进而改变免疫微环境的研究已经得到阐明。研究^[19]^表明，吉西他滨、紫杉醇和卡铂等化疗药物可通过激活NF-κB信号通路诱导PD-L1上调。一项特瑞普利单抗联合化疗治疗EGFR-TKIs失败且无T790M突变的晚期NSCLC患者的II期临床研究（NCT03513666）^[28]^，结果显示在40例意向治疗（intent to treat, ITT）人群中，特瑞普利单抗联合化疗ORR为50%，DCR为87.5%，mPFS和mOS分别为7.0个月和23.5个月。2021年ESMO免疫肿瘤大会上报道了替雷利珠单抗联合白蛋白紫杉醇和卡铂治疗靶向耐药的EGFR突变NSCLC II期临床研究^[19]^，结果显示其中32例患者ORR为59.4%（95%CI: 40.6%-76.3%），DCR为90.6%（95%CI: 75.0%-98.0%）。这两项研究^[19,28]^结果提示ICIs联合化疗针对EGFR-TKIs耐药患者具有良好临床获益，并且不良反应未有明显增加。ICIs联合化疗应对靶向耐药的探索并未停止，III期KEYNOTE-789（NCT03515837）以及CheckMate 722（NCT02864251）研究结果目前尚未公布，这两项研究将会进一步回答免疫联合化疗对靶向治疗耐药NSCLC患者作用如何。

### 4.3 ICIs联合抗血管±化疗治疗

抗血管生成和ICIs治疗具有相互协同的作用。一方面，抗血管生成治疗可以阻断负性免疫信号；另一方面，ICIs治疗可以恢复免疫支持的微环境进而促进血管正常化^[29]^。一项回顾性研究^[30]^比较帕博利珠单抗单药治疗（PM）、帕博利珠单抗联合化疗（P+C）和帕博利珠单抗联合安罗替尼（P+A）在TKIs和铂类化疗治疗失败的EGFR突变NSCLC患者中的临床疗效。共入组86例患者，研究结果提示PM组、P+C组及P+A组的mPFS分别为1.5个月、4.3个月及3.24个月，mOS分别为7.41个月、14.92个月及15.97个月，ORR分别为3.1%、23.1%及21.4%。一项IB期/II期研究（NCT03083041）^[31]^评估了卡瑞利珠单抗联合阿帕替尼治疗有EGFR或ALK基因突变的晚期NSCLC患者的疗效和安全性。入组的43例患者都接受至少一次EGFR/ALK-TKIs和以铂为基础的化疗方案。结果显示：ORR为18.6%（95%CI: 8.4%-33.4%），mPFS为2.8个月（95%CI: 1.9-5.5），未达到mOS（95%CI: 7.3-NR），中位随访期为15.7个月（95%CI: 0.5-24.4）。该研究提示卡瑞利珠单抗联合阿帕替尼在该群体患者中显示出中等的抗肿瘤活性和可接受的安全性，结果也值得进一步验证。IMpower150研究^[32]^是旨在评估阿替利珠单抗（A）+卡铂（C）+紫杉醇（P）联合或不联合贝伐珠单抗（B）的疗效及安全性的一项随机对照III期临床试验，其中亚组分析结果表明，在经TKIs治疗过的EGFR敏感突变的患者中，ABCP组mOS（27.8个月）长于BCP组（18.1个月）；ABCP vs BCP的HR点估计值为0.74（95%CI: 0.38-1.46）。尽管研究数据多数来自于探索性分析或单臂小样本研究，缺乏进一步的随机对照研究来佐证，但是ABCP四药联合为既往TKIs治疗失败的EGFR敏感突变这部分亚组患者带来了生存获益。ORIENT-31研究^[33]^是全球首个针对EGFR-TKIs治疗失败的EGFR突变非鳞NSCLC人群的随机对照、双盲、多中心的III期研究，前瞻性探索了信迪利单抗联合或不联合贝伐珠单抗及化疗治疗这一特定人群的临床疗效和安全性。期中分析的第二次结果提示，信迪利单抗联合贝伐珠单抗及化疗组（A组）、信迪利单抗联合化疗组（B组）以及化疗组（C组）的mPFS分别为7.2个月、5.5个月及4.3个月，ORR分别为48.1%、34.8%及29.4%。安全性方面，A组的总体安全性可控可接受，与B组相比仅高血压及蛋白尿不良事件发生率略有升高，但未明显增加总体不良事件发生率，患者能够较好耐受。BGB-A317-2001-IIT拓展计划是一项替雷利珠单抗联合贝伐珠单抗+化疗治疗伴EGFR敏感突变且既往EGFR-TKCs治疗失败的非鳞NSCLC患者的疗效及安全性的临床研究。目前该研究正在进行中，期待后续的研究结果。这些研究提示相比传统化疗方案，ICIs联合抗血管及化疗方案治疗晚期TKIs耐药患者的mPFS均能获得显著生存获益，为临床实践提供更多选择的方案（表1）。

**表1:** EGFR-TKIs耐药后ICIs联合治疗部分研究汇总

Item	Study	n	Phase	Primary end point	Treatment plan	ORR	mPFS (mon)	Security
ICIs+TKIs	CheckMate 012	20	I	Security	Nivolumab+Erlotinib	15%	5.1	≥G3 TRAEs: 24%; ≥irAEs: 36.7%; TEAEs causes treatment interruption: 6.7%
	GEFTREM terminate	27	I	Security	Tremelimumab+Gefitinib	0	2.2	≥G3 TRAEs: 81%
	Tatton suspension of recruitment	34	IB	Security	Durvalumab+Osimertinib	/	/	ILD: 35%
	CAURAL suspension of recruitment	29	III	Security	Durvalumab+Osimertinib; Osimertinib	80% vs 64%	/	ILD: 2%
ICIs+ Chemotherapy	2019 WCLC NCT03513666	40	II	ORR	Pembrolizumab+PEM/CBP	50.0%	7.0	≥G3 TRAEs: 65%; ≥irAEs: 5.0%; TEAEs caused treatment interruption: 10.0%
	2021 ESMO	32	II	PFS	Tislelizumab+NAB-PTX+CBP	59.4% (95%CI: 40.6%-76.3%)	/	≥G3 TRAEs: 32.5%; SAEs: 17.5%
	KEYNOTE789 be in progress	/	III	OS	Pembrolizumab+PEM+CBP; PEM+CBP	/	/	/
	CheckMate 722 be in progress	/	III	PFS	Nivolumab+Chemotherapy; Chemotherapy	/	/	/
ICIs+ Chemotherapy+ Antiangiogenic therapy	2020 ESMO IMpower150	79 ABCP vs BCP 34 vs 44	III subgroup	OS	Atezolizumab+PTX+CBP+BEV; PTX+CBP+BEV	73.5% vs 40.9%	10.2 vs 7.1; HR=0.56 (95%CI: 0.34-0.91)	≥G3 TRAEs: 66.7% vs 55.8%; SAEs: 42.4% vs 20.9%
	2020 WCLC NCT03083041	43	IB/II	PFS	Camrelizumab+Apatinib	18.6% (95%CI: 8.4%-33.4%)	2.8 (95%CI: 1.9-5.5)	/
	2021 ESMO ASIA ORIENT-31	299 148 vs 151	III	PFS	Sintilimab+PEM+DDP+BEV; PEM+DDP	48.1% vs 29.4%	7.2 vs 4.3; HR=0.464 (95%CI: 0.34-0.91)	≥G3 TRAEs: 54.7% vs 51%; SAEs: 37.2% vs 35%; TEAEs caused treatment: 19.9% vs 6.6%; irAEs: 35.1% vs 15.2%
	BGB-A317-2001-IIT be in progress	32	II	PFS	Tislelizumab+NAB-PTX+BEV	/	/	/

EGFR: epidermal growth factor receptor; TKIs: tyrosine kinase inhibitors; ICIs: immune checkpoint inhibitors; ORR: objective response rate; PFS: progression-free survival; OS: overall survival; TRAEs: treatment-related adverse events; WCLC: World Conference on Lung Cancer; ESMO: European Society of Medical Oncology; irAEs: immune-related adverse events; SAEs: serious adverse events; ILD: interstitial lung disease; PEM: Pemetrexed disodium; CBP: Carboplatin; NAB-PTX: Paclitaxel protein-bound; BEV: Bevacizumab; DDP: Cisplatin; /: no data.

### 4.4 ICIs联合ICIs

目前关于两种ICIs联合治疗EGFR突变患者研究数据尚不足。在CheckMate 012研究^[34]^中，其中8例未曾接受化疗治疗的EGFR突变经靶向治疗耐药后的患者接受了纳武利尤单抗联合伊匹木单抗，ORR为50%。NCT03091491^[35]^是一项3个临床中心进行的随机II期临床试验，旨在评估纳武利尤单抗单药对比纳武利尤单抗联合伊匹木单抗治疗靶向治疗耐药后的EGFR突变NSCLC患者的疗效，单药组出现PD后允许交叉到联药组。研究结果显示：共30例患者可供评估，仅联药组有1例患者PR。两组患者的PFS没有差异，总体队列mPFS为1.22个月（95%CI: 1.15-1.35），mOS为5.56个月（95%CI: 3.81-10.59）。5例患者获得生存获益，均为无烟史和ex19del突变，其中1例T790M阳性，1例PD-L1>50%。目前临床研究提示双免治疗可能不足以在治疗EGFR-TKIs耐药的NSCLC患者中获得临床益处，是否存在其他双免药物联合治疗模式可以给这部分人群带来获益还需进一步探索。

## 5 新型ICIs

根据T细胞靶向免疫调节剂免疫学，克服ICIs效率低下的另一种有希望的治疗方法是针对与TME相关的其他ICIs。一些针对NSCLC患者的新型ICIs，如淋巴细胞活化因子3（lymphocyte activation gene 3, LAG3）、T细胞免疫球蛋白ITIM结构域（T cell immunoglobulin and ITIM domains, TIGIT）和B7同源物3（B7 nomolog 3, B7H3）的临床研究正在进行。有报道^[36]^称，在EGFR突变的NSCLC患者中，经过TKIs治疗耐药后LAG3会发生上调，为这部分患者抗LAG治疗提供了新见解。在探索新型ICIs单一或联合治疗可能为TKIs耐药后EGFR突变的NSCLC患者提供更多和更好的治疗选择。

## 6 EGFR突变晚期NSCLC免疫治疗潜在获益人群

EGFR野生型NSCLC中判定预后的PD-L1表达、TMB、CD8^+^肿瘤浸润淋巴细胞（tumor infiltrating lymphocytes, TILs）等生物标志物，在EGFR突变晚期NSCLC并不能完全适用，一些伴有EGFR突变的NSCLC患者确实对ICIs有反应，但潜在受益人群的特征仍不清楚。相关研究^[37]^表明，伴有常见EGFR突变相比伴有罕见EGFR突变晚期NSCLC患者对ICIs的反应更差。这可能是因为伴有罕见EGFR突变的NSCLC患者有更高水平的PD-L1表达和更丰富的CD8^+ ^TILs。为了比较ICIs对L858R和ex19del的疗效，一项研究^[38]^纳入EGFR突变的NSCLC患者，在对EGFR-TKIs耐药后单独应用ICIs或联合治疗，发现EGFR L858R突变患者的mPFS和mOS都明显短于EGFR ex19del的患者（mPFS：2.5个月 vs 6.7个月，HR=1.80，P=0.011；mOS：9.8个月 vs 26个月，HR=2.84，P=0.002）。但另一项研究^[32]^纳入EGFR突变晚期NSCLC单独应用ICIs或联合细胞毒性T淋巴细胞相关抗原4（cytotoxic T-lymphocyte-associated antigen 4, CTLA-4）抑制剂，结果显示EGFR ex19del突变ORR较野生型显著降低（7% vs 22%, P=0.002），PFS及OS均缩短。可能因为EGFR L858R突变患者具有较高TMB，这部分患者的ORR（16% vs 22%, P=0.42）和OS较野生型无明显差异。对于T790M状态，Inomata等^[39]^发现在T790M阴性的患者中，PD-L1强阳性的比例高于T790M阳性的患者（P=0.022），经过随后的免疫治疗，T790M阴性机制介导的获得性耐药患者的PFS也高于T790M阳性患者（16.5个月 vs 8.6个月，P=0.001）。同样关于EGFR突变阳性NSCLC患者在EGFR-TKIs失败后使用纳武利尤单抗疗效的回顾性研究^[4]^发现，T790M阴性患者比T790M阳性患者更可能从纳武利尤单抗中获益。TP53作为影响EGFR靶向治疗疗效的常见因素之一，同样可以影响患者使用免疫治疗的疗效。TP53突变或缺失将导致细胞周期紊乱，抑制细胞凋亡，影响DNA损伤修复功能从而导致基因组不稳定。同时研究^[40]^发现TP53突变增加肿瘤PD-L1表达，这些都可能增加TMB，进而具有更好的免疫治疗疗效。

因此，基于ICIs联合治疗是EGFR-TKIs耐药后EGFR突变的NSCLC患者潜在有效治疗策略，其疗效可能因EGFR亚型不同而不同。似乎罕见突变相较常见突变对于ICIs治疗反应更好，ICIs对于常见突变L858R及ex19del的疗效研究结果还存在争议，这可能与研究样本量及具体治疗方案相关，仍需进一步探索。T790M及TP53突变同样可以从一定程度预测这部分患者免疫治疗的疗效。

## 7 免疫联合治疗相关不良反应

尽管PD-1及PD-L1单一疗法通常耐受性良好，但两项荟萃分析^[41,42]^表明，PD-1和PD-L1与CTLA-4联合治疗的不良事件发生率显著高于PD-1或PD-L1单药治疗。关注ICIs联合治疗的不良反应发生情况，可以指导临床医生更好地评估治疗风险和管理潜在的不良反应。

一项关于包括161项研究的荟萃分析^[43]^旨在考察PD-1和PD-L1抑制剂联合治疗中TRAEs的发生率情况，结果显示：联合治疗的不良反应相比ICIs单药发生率更高，包括疲劳、发热、腹泻、皮肤毒性、甲状腺功能障碍、肺炎、肌酐升高和脂肪酶升高。但有几种不良事件值得注意，在CTLA-4抑制剂联合治疗中，脑垂体炎是一种比单一治疗更常见的不良反应。CTLA-4在脑垂体中的异位表达可导致直接的非靶点损伤，垂体功能在使用CTLA-4抑制剂联合治疗时应特别关注。血液学事件是对于接受PD-1或PD-L1抑制剂联合化疗治疗的患者主要的不良事件，这在很大程度上归因于化疗药物的细胞毒性。血管内皮生长因子（vascular endothelial growth factor, VEGF）和VEGF受体（VEGF receptor, VEGFR）抑制剂联合应用可特异性地引起高血压和蛋白尿，这可能与其抗血管生成机制有关^[43]^。了解TRAEs提高临床医生的认识，从而做到常规筛查和早期治疗，对于患者生存获益至关重要。

## 8 结语与展望

免疫治疗改变了晚期NSCLC的治疗模式，也为EGFR-TKIs耐药患者带来了新的希望。但多项研究^[11,18,34,35]^数据表明ICIs单药与双免联合治疗对于这部分患者未有明显临床获益，ICIs与抗血管生成药物和化疗的联合应用可能是未来很有前途的研究方向。如何应用多种生物标志物综合评价从而筛选潜在获益人群并更为精准地预测这部分人群的免疫治疗疗效，制定合理的治疗方案，减少不良反应的发生，是未来的重点研究方向。
